# System Vicarious Calibration and Atmospheric Correction of the Airborne Watersat Imaging Spectrometer Experiment

**DOI:** 10.3390/s26185689

**Published:** 2026-09-08

**Authors:** Raphael Mabit, Simon Bélanger

**Affiliations:** 1 Institut des Sciences de la Mer de Rimouski, Université du Québec à Rimouski (UQAR), 310 Allée des Ursulines, Rimouski, QC G5L 3A1, Canada; 2 Québec-Océan: Groupe Interinstitutionnel de Recherches Océanographiques du Québec, Quebec City, QC G1V 0A6, Canada; 3 Département de Biologie, Chimie et Géographie, Université du Québec à Rimouski (UQAR), 300 Allée des Ursulines, Quebec City, QC G5L 3A1, Canada

**Keywords:** ocean color, remote sensing, vicarious calibration, atmospheric correction

## Abstract

We present the vicarious calibration and validation of atmospheric correction for the airborne Watersat Imaging Spectrometer Experiment (WISE) sensor, deployed over the south-west shores of Anticosti Island (Gulf of Saint Lawrence). Coincident in situ water-leaving reflectance was acquired from the research vessel *Coriolis II* (HyperSAS, above-water radiometry) and from a jet-ski (underwater radiometry). The HyperSAS Hyperspectral Surface Acquisition System dataset was used for calibration, while the jet-ski dataset was split into 30% calibration and 70% validation. We evaluated Ratio and Empirical Line (EL) calibration approaches and investigated the influence of residual sun glint. Modeled vs. measured at-sensor reflectance residuals show a clear dependence on angular distance to the specular direction, demonstrating that geometry-dependent glint bias affects vicarious gain estimation. The Ratio approach performs adequately with a limited calibration range (∼50% accuracy at 500 nm) but degrades when applied to a broader matchup dataset. In contrast, the EL method provides more stable gains by accommodating both additive and multiplicative discrepancies (∼20% accuracy at 500 nm). These results highlight the importance of accounting for residual sun glint in airborne aquatic imaging spectroscopy (for calibration or other purposes) and support the EL with Standard Major Axis regression as the most robust calibration strategy for quantitative retrieval of water-leaving reflectance from WISE.

## 1. Introduction

Imaging spectroscopy is a powerful tool to study natural environments: with high spatial and spectral resolution, it is possible to detect features with fine spatial and spectral signatures. To use imaging spectroscopy to its full potential, one must ensure that the reflectance products derived after atmospheric correction have high precision and accuracy [1], both in terms of spectral shape and magnitude. This is particularly challenging for aquatic applications, where the contribution of water-leaving signal to the total signal recorded from an airborne or spaceborne platform is relatively small. The Watersat Imaging Spectrometer Experiment (WISE) sensor, a prototype developed by ITRES under contract with the Canadian Space Agency, presents an enhanced signal-to-noise ratio in the visible part of the spectrum relative to its predecessor, the Compact Airborne Spectrographic Imager (CASI). Extensive characterization and testing of WISE conducted by the Flight Research Laboratory of the National Research Council revealed an issue affecting the absolute radiance calibration when applied to airborne imagery. The measured at-sensor radiance $L_{t}$ was systematically underestimated, preventing the direct application of atmospheric correction required for accurate surface reflectance retrieval [2]. The WISE sensor was deployed in July 2022 over Baie Gamache on Anticosti Island in the Gulf of St. Lawrence, coincident with an extensive set of in situ radiometric measurements. During the processing of the WISE imagery, an initial calibration was applied using known surface reflectance of land targets together with the ATCOR (Atmospheric and Topographic Correction) atmospheric correction model [3]. The resulting surface reflectance product showed good agreement with the measured land reflectance. However, it was not suitable for water color remote sensing applications, as additional corrections for sky and sun glint on the water surface were needed.

Several methods have been proposed in the literature to convert sensor-measured signals (raw counts or calibrated radiance) into surface reflectance. The simplest approach is the Empirical Line (EL), which consists of performing a linear regression between the remote-sensor signal and the surface reflectance of at least one dark and one bright target [4,5]. Other approaches derive calibration coefficients directly from contrasting targets within the image, such as the coastal tree-shadow method [6]. When no in situ reflectance measurements are available, atmospheric correction methods can be applied to retrieve water-leaving radiance or reflectance from at-sensor radiance. Nevertheless, even these methods benefit from calibration using in situ reflectance. Numerous airborne and spaceborne imaging spectrometers have demonstrated the need for system vicarious calibration (SVC) to achieve the radiometric accuracy required for aquatic remote-sensing applications. Because water-leaving radiance represents only a small fraction of the total at-sensor signal (often less than 10% in the visible domain; Gordon and Clark [7]), small biases in absolute radiometric calibration or in the atmospheric-correction model can lead to significant errors in retrieved reflectance. For instance, both the Airborne Visible/Infrared Imaging Spectrometer (AVIRIS) [8] and Portable Remote Imaging SpectroMeter (PRISM) [9] required radiometric adjustment procedures to meet the accuracy thresholds necessary for quantitative water applications [10].

In this study, we focus on retrieving water-leaving reflectance ($\rho_{w}$, dimensionless). First, we adapted the Dark Spectrum Fitting (DSF) atmospheric correction algorithm [11] for application to airborne WISE imagery. Second, to ensure retrieval of high-quality water-leaving reflectance, we evaluated two vicarious-calibration methods based on the relationship between the modeled and observed at-sensor reflectances (respectively, ${\hat{\rho }}_{t}$ and $\rho_{t}$). To demonstrate the usefulness of the methods, we present the retrieved $\rho_{w}$ both before and after the application of the vicarious corrections. Finally, the end product of this vicarious calibration workflow is a complete dataset of $\rho_{w}$ imagery ready for aquatic applications, including the mapping of subtidal habitats and the characterization of surface-water optical properties in the southwestern shores of Anticosti Island.

## 2. Materials and Methods

### 2.1. Algae-WISE Fieldwork

**Figure 1:**
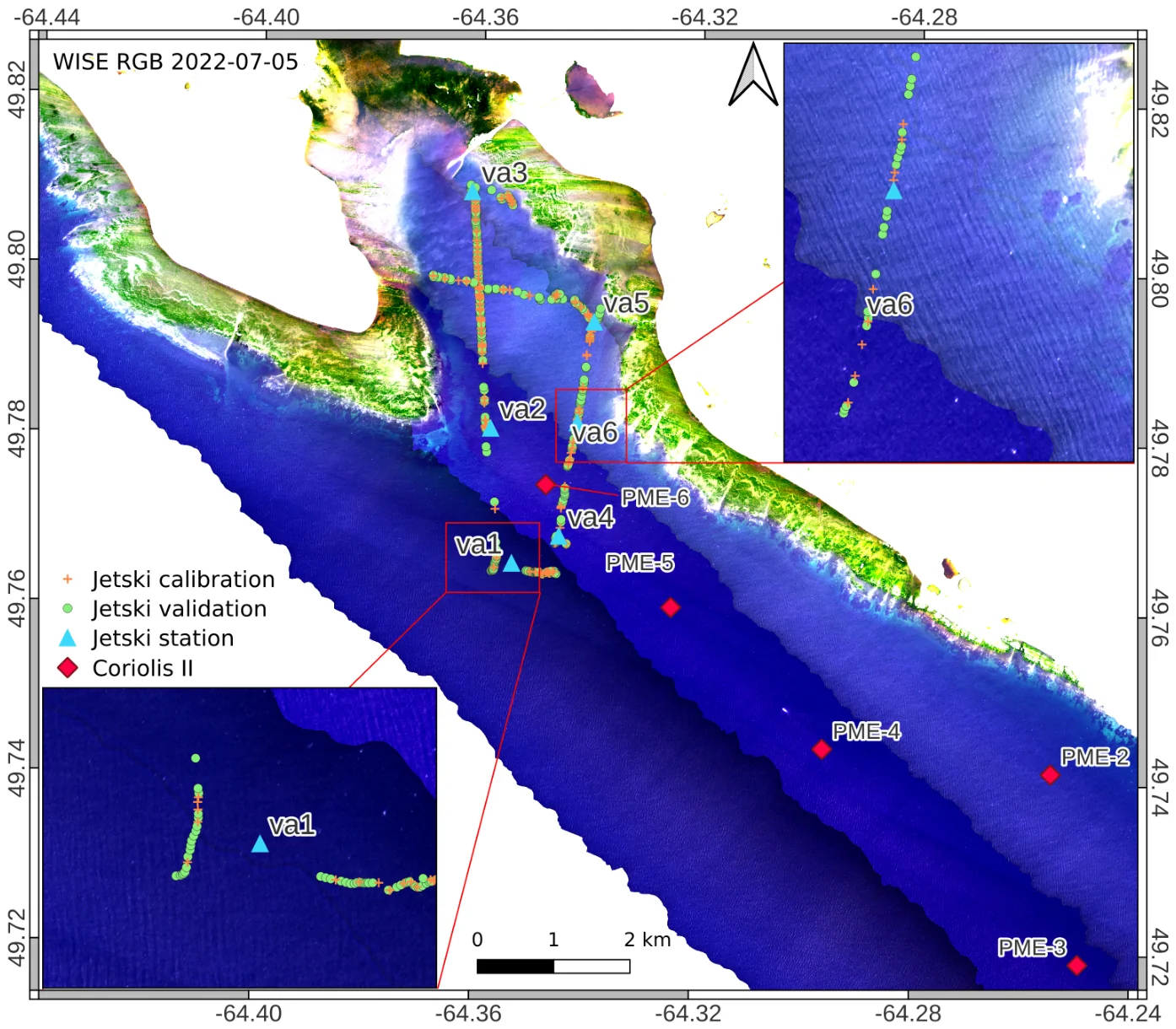
Data localization and true-color RGB composite of water leaving reflectance from WISE.

The Algae-WISE fieldwork aimed to deploy the WISE sensor over the south-western shores of Anticosti Island, coincident with an extensive set of in situ measurements (Figure 1). The waters around Anticosti Island are generally clear (∼10 m visibility) and host extensive kelp beds from the shore to ∼20 m depth. The WISE sensor is a pushbroom airborne hyperspectral sensor with a 20-degree field of view. It was configured with 144 spectral channels (out of 288 measured channels) and a 32 ms integration time. The spectral bands cover the range from 361 nm to 991 nm with a spectral resolution of approximately 5 nm. Flight lines were acquired at an altitude of about 3000 m, yielding a ground sampling distance of roughly 1.5 m along track (for more technical details about the WISE sensor see [2]). The in situ data collected for this study were described in [12]. Two platforms for in-water and above-water radiometric measurements were deployed near Baie Gamache: the R/V *Coriolis II* and a jet-ski.

The *Coriolis II* was equipped with a HyperSAS to estimate water-leaving reflectance from above-water radiometry, following the recommended protocol [13]. The *Coriolis II* acquired data at five stations inside the study area. The jet-ski is a continuous acquisition platform (i.e., acquiring data while underway) equipped with two in-water upwelling radiance HOCRs (hyperspectral ocean color radiometers) at different depths and an above-water downwelling irradiance HOCR. The continuous acquisition ability is what enables us to acquire a dataset of in situ water-leaving reflectance covering the entire range of viewing zenith angle of the WISE sensor. The jetski can also be used to perform stationary sampling. To represent the retrieved water-leaving reflectance spectra obtained using the different calibration techniques, we selected six in situ water-leaving reflectance matchups with the flight-line images. Those spectra are associated with stations va1 to va6 shown in Figure 1. For additional detail on the in situ measurement platforms and methodology, the reader is referred to [12].

### 2.2. Atmospheric Correction Algorithm

The so-called “apparent” reflectance signal at the altitude of the sensor $\rho_{t}$ is defined as

(1) $$\rho_{t}=\frac{L_{t}\pi }{F_{0}^{\prime }\cos\theta_{s}}$$

where $L_{t}$ is the at-sensor radiance, $\theta_{s}$ is the sun zenith angle, and $F_{0}$ is the extraterrestrial solar irradiance from [14] computed for a specific day as $F_{0}^{\prime }=F_{0}\left(1+0.34\cos\left(2\pi \left(J-2\right)/365\right)\right)$ [15], with *J* being the day of the year.

The at-sensor reflectance can be expressed as a contribution of surface and path reflectance, assuming a Lambertian surface [16],

(2) $$\rho_{t}=t_{g}\left(\rho_{\mathrm{path}}+t_{\mathrm{ra}}\frac{\rho_{s}}{1-s_{\mathrm{ra}}\rho_{s}}\right),$$

where $t_{g}$ is the gas transmittance, $\rho_{\mathrm{path}}$ is the path reflectance intrinsic to the molecule + aerosol layer, $t_{\mathrm{ra}}$ is the two-way diffuse atmospheric transmittance due to molecule+aerosols and $s_{\mathrm{ra}}$ is the spherical albedo of the atmospheric gases and aerosols, and $\rho_{s}$ is the surface reflectance. From Equation (2), the at-sensor total signal can be corrected for the effect of the atmosphere to estimate the surface reflectance:

(3) $$\rho_{s}=\frac{\rho_{t}/t_{g}-\rho_{\mathrm{path}}}{t_{\mathrm{ra}}+s_{\mathrm{ra}}\left(\rho_{t}/t_{g}-\rho_{\mathrm{path}}\right)}.$$

To retrieve the water-leaving signal, the diffuse sky-glint on the sea surface $\rho_{\mathrm{sky}\text{-}\mathrm{glint}}$ is then subtracted:

(4) $$\rho_{w}=\rho_{s}-\rho_{\mathrm{sky}\text{-}\mathrm{glint}}.$$

We generated LUTs of atmospheric and surface parameters computed with the 6S model [16]. Specifically, two LUTs were produced: one for gas containing $t_{g}$, and another for aerosol (using the maritime aerosol model) containing diffuse transmittance $t_{\mathrm{ra}}$, $\rho_{\mathrm{path}}$, $s_{\mathrm{ra}}$, and $\rho_{\mathrm{sky}\text{-}\mathrm{glint}}$. The sky glint was computed according to the following [11,17]:

(5) $$\rho_{\mathrm{sky}\text{-}\mathrm{glint}}=\tau_{\mathrm{ra}}p_{\mathrm{ra}}/\left(4\cos\theta_{s}\cos\theta_{v}\right),$$

where $\tau_{\mathrm{ra}}$ is the optical depth of Rayleigh and aerosol, $p_{\mathrm{ra}}$ is the scattering phase function of Rayleigh and aerosol scaled by the Fresnel reflectance, and $\theta_{v}$ is the viewing zenith angle.

The modifications introduced to the DSF algorithm [11] include the addition of the sensor altitude as a dimension in the LUT. In this study, we constructed the dark spectrum ($\rho_{\mathrm{dark}}$) by taking the first percentile of $\rho_{t}$ in each spectral channel. This dark spectrum is assumed to represent the path reflectance ($\rho_{\mathrm{path}}$), which is considered to originate exclusively from the atmosphere. Although $\tau_{a}\left(555\right)$ is defined at a fixed wavelength, it can be estimated from any spectral channel because the LUT provides the expected $\rho_{\mathrm{path}}$ spectrum for a given $\tau_{a}\left(555\right)$. Consequently, each wavelength of $\rho_{\mathrm{path}}$ can be interpolated to $\rho_{\mathrm{dark}}$ and provide an independent estimate of $\tau_{a}\left(555\right)$. The minimum value of $\tau_{a}\left(555\right)$ over the spectrum is retained for the final correction [11], avoiding negative water-leaving reflectance retrievals.

A final correction was needed for residual glint, which assumes that $\rho_{w}$ is zero at a near-infrared (NIR) reference wavelength (here at 800 nm; $\rho_{w}\left(800\right)$) [18],

(6) $$\rho_{w}\left(G21\right)=\rho_{w}-\left(\rho_{\mathrm{fresnel}}\frac{\rho_{w}\left(800\right)}{\rho_{\mathrm{fresnel}}\left(800\right)}\right)$$

where $\rho_{w}\left(G21\right)$ is the Gao and Li (2021) corrected water-leaving reflectance, and $\rho_{\mathrm{fresnel}}$ is the Fresnel reflectance computed at nadir, as we cannot know a priori the angle of the sun on the wave slope [18]. The assumption of zero water-leaving reflectance in the NIR may break down in turbid coastal waters or in the presence of floating vegetation, which would generate a non-negligible signal at those wavelengths. In that case, the NIR signal will be overcorrected, leading to erroneous $\rho_{w}$ values. Therefore, depending on the vicarious calibration method applied, we report either $\rho_{w}\left(G21\right)$ or $\rho_{w}$ as the most realistic estimate.

### 2.3. Vicarious Calibration

The vicarious calibration procedure is the reciprocal of the atmospheric correction [1]. This procedure is often called system vicarious calibration because it allows for correction of both the discrepancy between laboratory calibration and airborne deployment and the errors introduced by the atmospheric correction model. The goal is to propagate, using the atmospheric correction model, an in situ surface reflectance at the airborne sensor altitude to simulate what the sensor should theoretically measure using Equation (2).

For the vicarious calibration, $\tau_{a}\left(555\right)$ is not retrieved from the image but taken from the MERRA2 atmospheric model [19]. To maintain consistency in the subsequent atmospheric correction applied to the recalibrated data, the MERRA2-derived $\tau_{a}\left(555\right)$ was used throughout.

We tested two vicarious calibration methods. First, the *Ratio* vicarious calibration method consists of dividing the modelled at-sensor reflectance (${\hat{\rho }}_{t}$) by the observed at-sensor reflectance, i.e., $\mathrm{Slope}=\frac{{\hat{\rho }}_{t}}{\rho_{t}}$ [1]. The slope multiplicative coefficient can then be applied to $\rho_{t}$ prior to atmospheric correction, ensuring that the sensor-corrected data would retrieve the same $\rho_{w}$ used as input to the forward model. Second, the *Empirical Line* method (EL) uses a linear model of the form ${\hat{\rho }}_{t}∼a\rho_{t}+b$ [2,20], where the coefficients *a* and *b* are obtained by fitting at least two targets with contrasting reflectance. The fit of the *EL* was obtained using Ordinary Least Squares (referred to as EL OLS) and Standard Major Axis ([21], referred to as SMA) to test the effect of accounting for uncertainties in the predictor and predicted variables (e.g., uncertainties in the water leaving reflectance and atmospheric model). We note that the *Ratio* vicarious calibration is equivalent to the *OLS* with the intercept (*b*) constrained to 0.

Two in situ datasets were used for the vicarious calibration. The first consists of five hyperspectral above-water reflectance measurements collected with a HyperSAS system deployed from the *R/V Coriolis II* (Figure 1). Because the HyperSAS spectral range (350–747 nm) does not extend to the NIR, it was complemented using a linear interpolation between in-water reflectance values measured with a C-OPS at 780 and 875 nm. This procedure allowed us to derive vicarious calibration gains across the 375–800 nm spectral range. The HyperSAS dataset (N = 5) was used to evaluate the Ratio calibration, referred to as *Ratio SAS*. Since the *EL* relies on regression, we anticipated that it could benefit from a larger number of observations spanning a broader range of $\rho_{w}$ values. For this reason, we randomly partitioned the jetski dataset into 30% calibration (N = 116) and 70% validation (N = 270). As we expect the range of $\rho_{w}$ to have a larger impact on calibration than the method used to compute it, the HyperSAS and jetski datasets are combined to evaluate the *Ratio SAS-jetski*, *EL OLS* and *EL SMA* vicarious calibrations.

To assess the potential impact of sun glint on the vicarious calibration process, we computed the angular distance between the viewing direction and the sun glint direction as

(7) $$\cos\gamma =\cos\theta_{v}\cos\theta_{s}+\sin\theta_{v}\sin\theta_{s}\cos\left(\Delta \phi -\pi \right)$$

where cosγ is the cosine of the angle between the viewing direction and the specular reflection of the sun; $\theta_{v}$, $\theta_{s}$ are the viewing and sun zenith, respectively; and Δϕ is the relative azimuth angle between the sun and the viewing direction. Smaller values of γ indicate that the sensor is viewing closer to the specular-reflection direction. However, this calculation assumes a flat, mirror-like ocean surface and does not account for surface-wave slope or orientation, but it nevertheless provides a useful diagnostic for interpreting our results.

### 2.4. Precision and Accuracy Metrics

To assess the precision and accuracy of the four vicarious calibration methods presented above, we use the Median Symmetric Accuracy (MdSA, Equation (8)) and the Symmetric Signed Percentage Bias (SSPB, Equation (9)) [22].

(8) $$\mathrm{MdSA}=100\left(10^{a}-1\right)\;\mathrm{where}\;a=\mathrm{median}\left|\log_{10}\left(\hat{y}/y\right)\right|$$

(9) $$\mathrm{SSPB}=100\mathrm{sign}\left(b\right)\left(10^{b}-1\right)\;\mathrm{where}\;b=\mathrm{median}\left(\log_{10}\left(\hat{y}/y\right)\right)$$

(10) $$\mathrm{MdAE}=\mathrm{median}|y-\hat{y}|$$

where $\hat{y}$ is the modeled quantity and *y* is the measured quantity.

Reporting both relative and absolute error metrics is advantageous. While MdSA and SSPB provide symmetric, scale-independent measures of relative performance, the median absolute error (MdAE) offers complementary insight—particularly in low-$\rho_{w}$ regimes where small absolute deviations can translate into disproportionately large relative errors.

## 3. Results

### 3.1. DSF Pre-Vicarious Calibration

**Figure 2:**
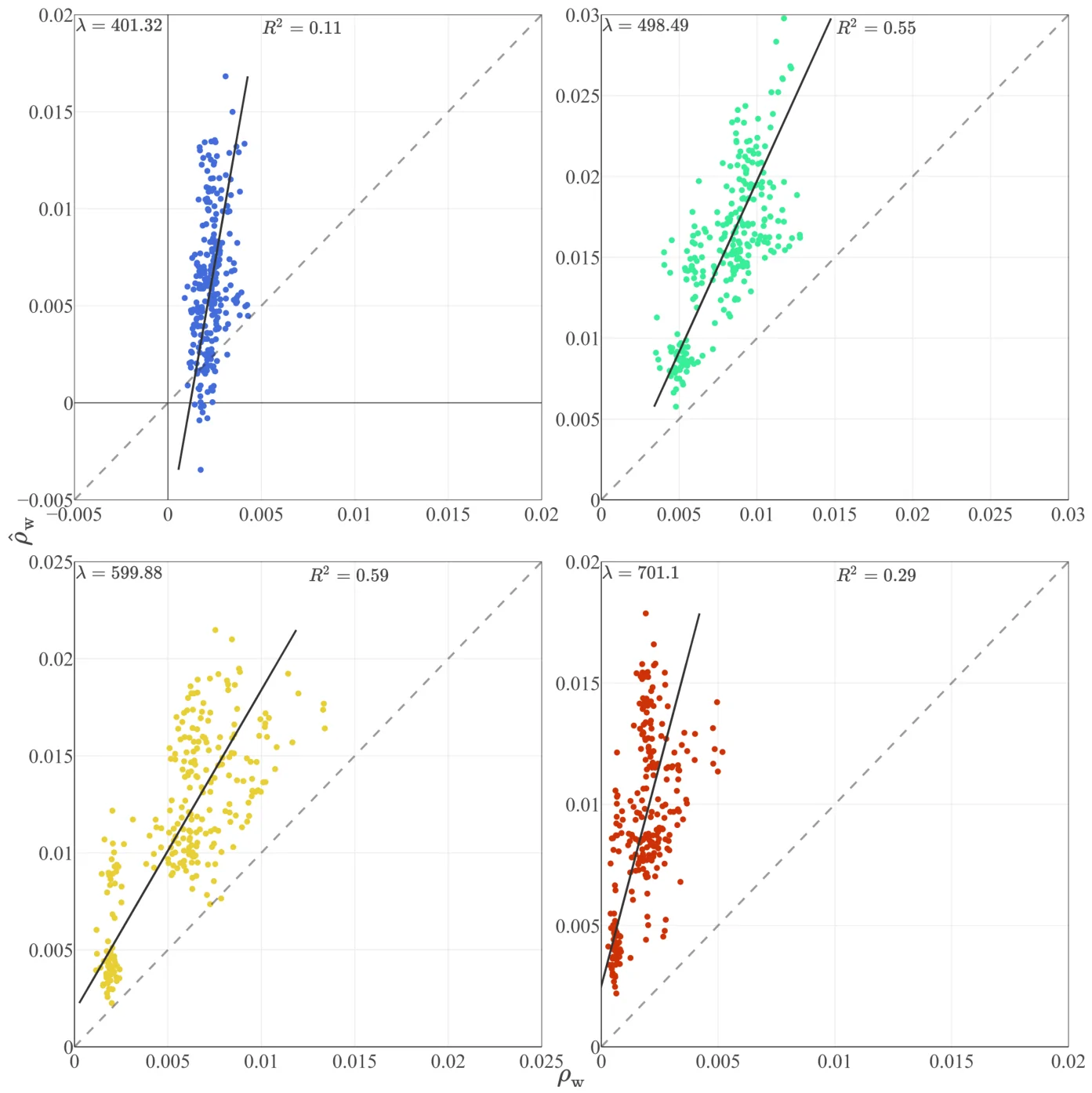
Water leaving reflectance retrieved from WISE DSF pre-vicarious calibration (${\hat{\rho }}_{w}$) compared to in situ $\rho_{w}$ from the jet-ski at four wavelengths. The dashed line correspond to the 1:1 line and the continous line to the linear regression between measured and retrieved water-leving reflectance.

To motivate the need for vicarious calibration, we first assess the performance of the DSF atmospheric correction applied to the land-target-based calibrated WISE at-sensor reflectance provided by the Flight Research Laboratory of the National Research Council. The pre-vicarious calibration retrieval yields some negative values of ${\hat{\rho }}_{w}$ values near 400 nm (Figure 2). At 400, 500, 600, and 700 nm, ${\hat{\rho }}_{w}$ shows a systematic overestimation relative to the jet-ski $\rho_{w}$, although the relationship remains strongly linear. We also note the wider dynamic range in the retrieved ${\hat{\rho }}_{w}$ relative to in situ $\rho_{w}$, which we attribute to the presence of residual sun glint in the airborne data.

**Figure 3:**
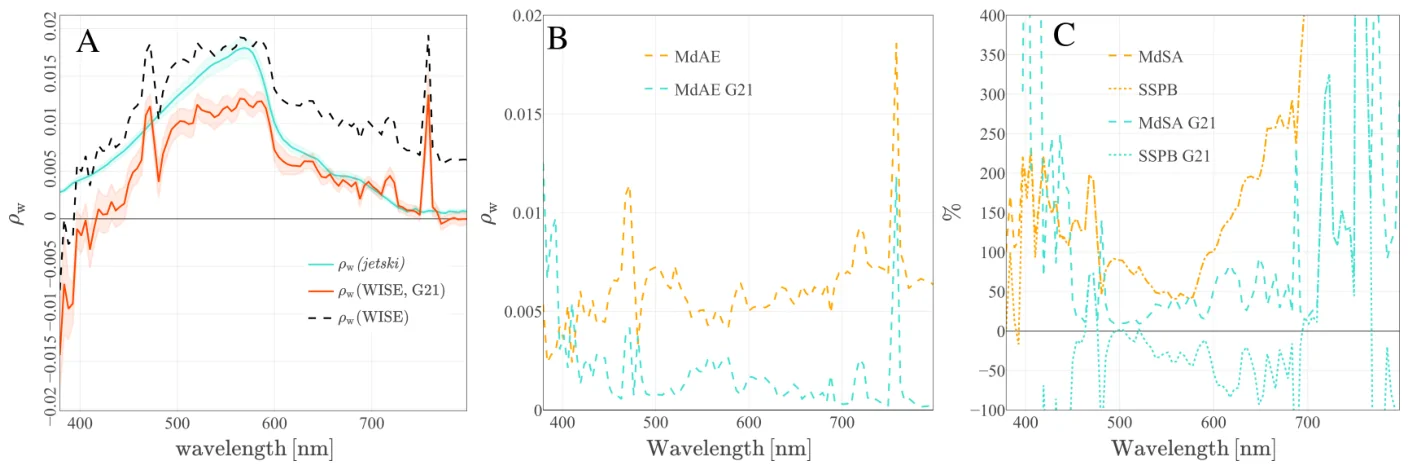
(**A**) WISE-derived ${\hat{\rho }}_{w}$ with and without glint correction (G21) compared to jetski $\rho_{w}$. (**B**) Absolute accuracy and (**C**) relative precision and accuracy of the DSF retrieved $\rho_{w}$ prior SVC.

The full spectra highlight the issue in the initial at-sensor reflectance more clearly (Figure 3). A relatively large positive bias is observed for wavelengths above 600 nm in ${\hat{\rho }}_{w}$ when no correction for residual sun glint is applied (black dashed line in Figure 3A). Applying the glint correction (G21) improves the retrieval in this spectral region, but introduces a general underestimation at shorter wavelengths, with negative values of ${\hat{\rho }}_{w}$ below ∼420 nm. Across the visible range, the pre-vicarious calibration product exhibits both spectral distortion and large band-dependent errors, supporting the need for a system vicarious calibration (SVC) before the data can be used for quantitative aquatic applications.

### 3.2. Calibration Gain

**Figure 4:**
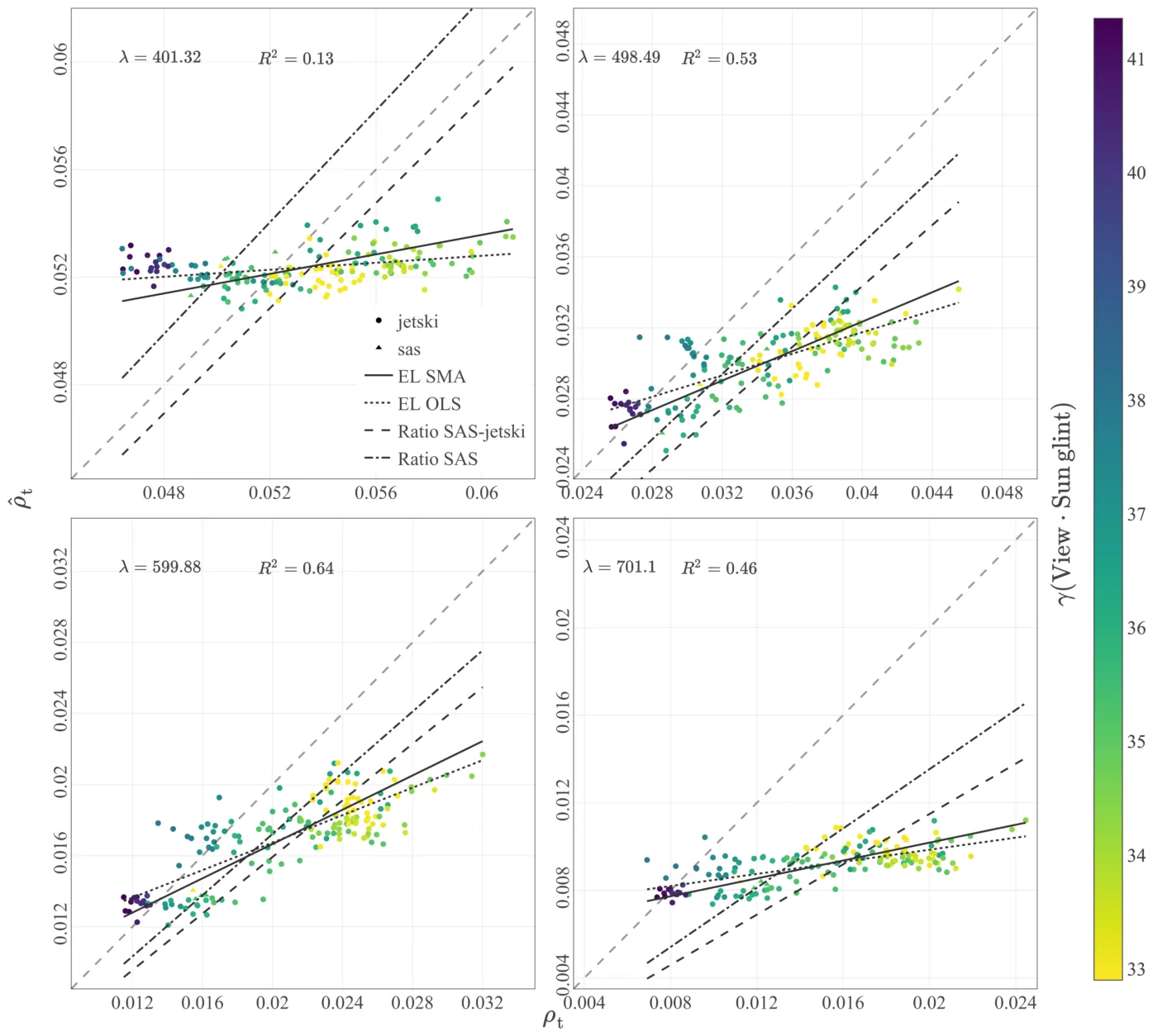
Modeled at-sensor reflectance (${\hat{\rho }}_{t}$) versus measured $\rho_{t}$ at four selected wavelengths. The color scale depicts the angle γ between the viewing direction and the sun’s specular reflection direction. The lines represent the calibration per method applied to $\rho_{t}$. $R^{2}$ is the squared correlation coefficient between $\rho_{t}$ and ${\hat{\rho }}_{t}$.

We next examine the relationship between modeled and measured at-sensor reflectance used to derive SVC gains. At selected wavelengths, the modeled-measured relationship shows limited sensitivity (i.e., a flat response) in the blue (400 nm) and red (700 nm) regions, consistent with the expected reduced dynamic range of $\rho_{w}$ (which is free of sun-glint contributions) and dominance of non-water in $\rho_{t}$ (Figure 4). The color scale in Figure 4 further illustrates how the measured $\rho_{t}$ is affected by sun glint, quantified here by the angle γ between the viewing direction and the specular-reflection direction of the sun.

**Figure 5:**
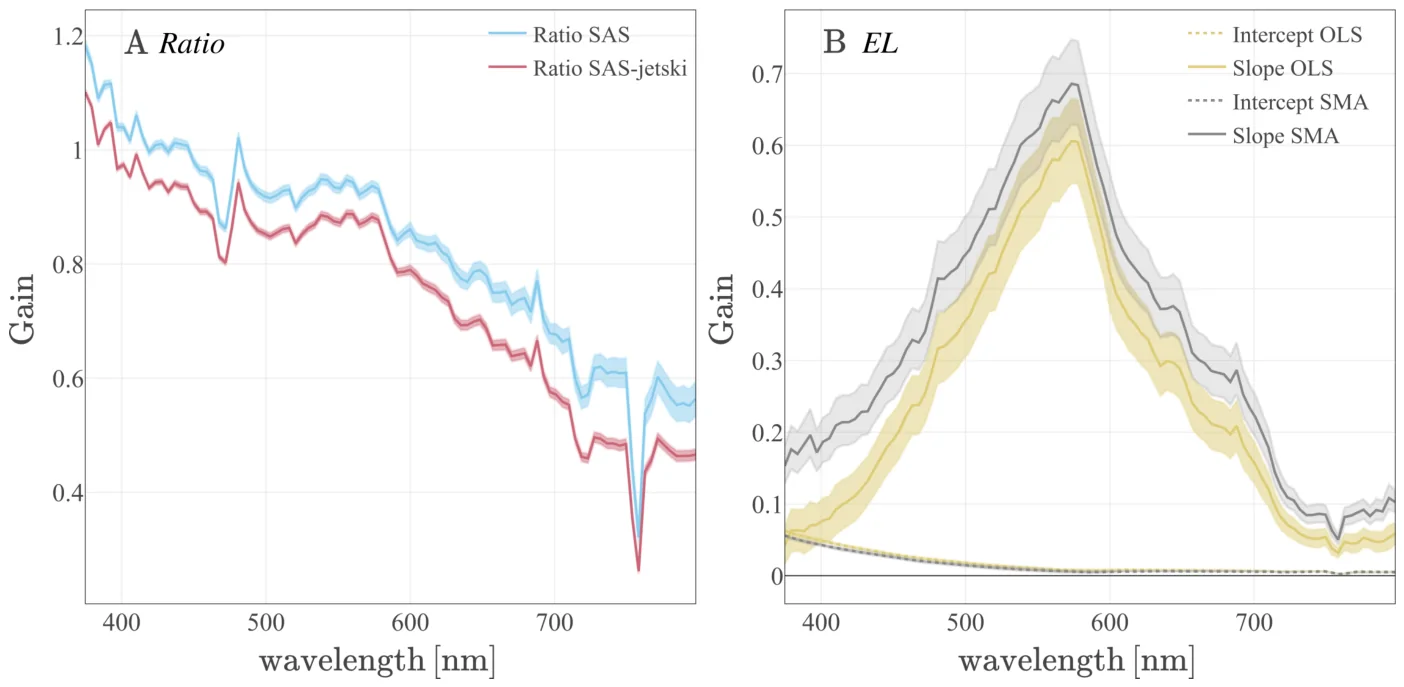
Vicarious calibration gain: (**A**) the *Ratio* method; (**B**) the *EL* using OLS (type I) and SMA (type II) regression methods. The continous lines represents the estimate of the parameters and the shaded area it’s uncertainty.

The linear model ${\hat{\rho }}_{t}∼a\rho_{t}+b$ produces a different spectral pattern (Figure 5B) from the *Ratio* gains (Figure 5A). The intercept term b(λ) decreases from blue to red and follows the expected spectral behavior of atmospheric path contributions (and/or sun glint), whereas the multiplicative coefficient a(λ) reflects the dominant wavelength-dependence of the water-leaving signal; for instance, the characteristic peak in the green near 560 nm.

The *Ratio* vicarious calibration slope is slightly above one below 400 nm, indicating a slight overestimation of the modeled at-sensor reflectance (${\hat{\rho }}_{t}$). Between 400 and 450 nm, the gain is close to unity, indicating a good agreement between the model and the observed $\rho_{t}$. Beyond 580 nm, the gain decreases steadily, interrupted only by a strong peak associated with the oxygen absorption band at 760 nm. Lower gains in this region indicate a substantial underestimation of ${\hat{\rho }}_{t}$.

Calibration gain computed for the merged jet-ski and HyperSAS dataset shows that the difference in the *Ratio* calibration arises primarily from the range of measured $\rho_{t}$ values, with sun glint likely accounting for an important portion of the observed variability (Figure 4, color scale).

**Figure 6:**
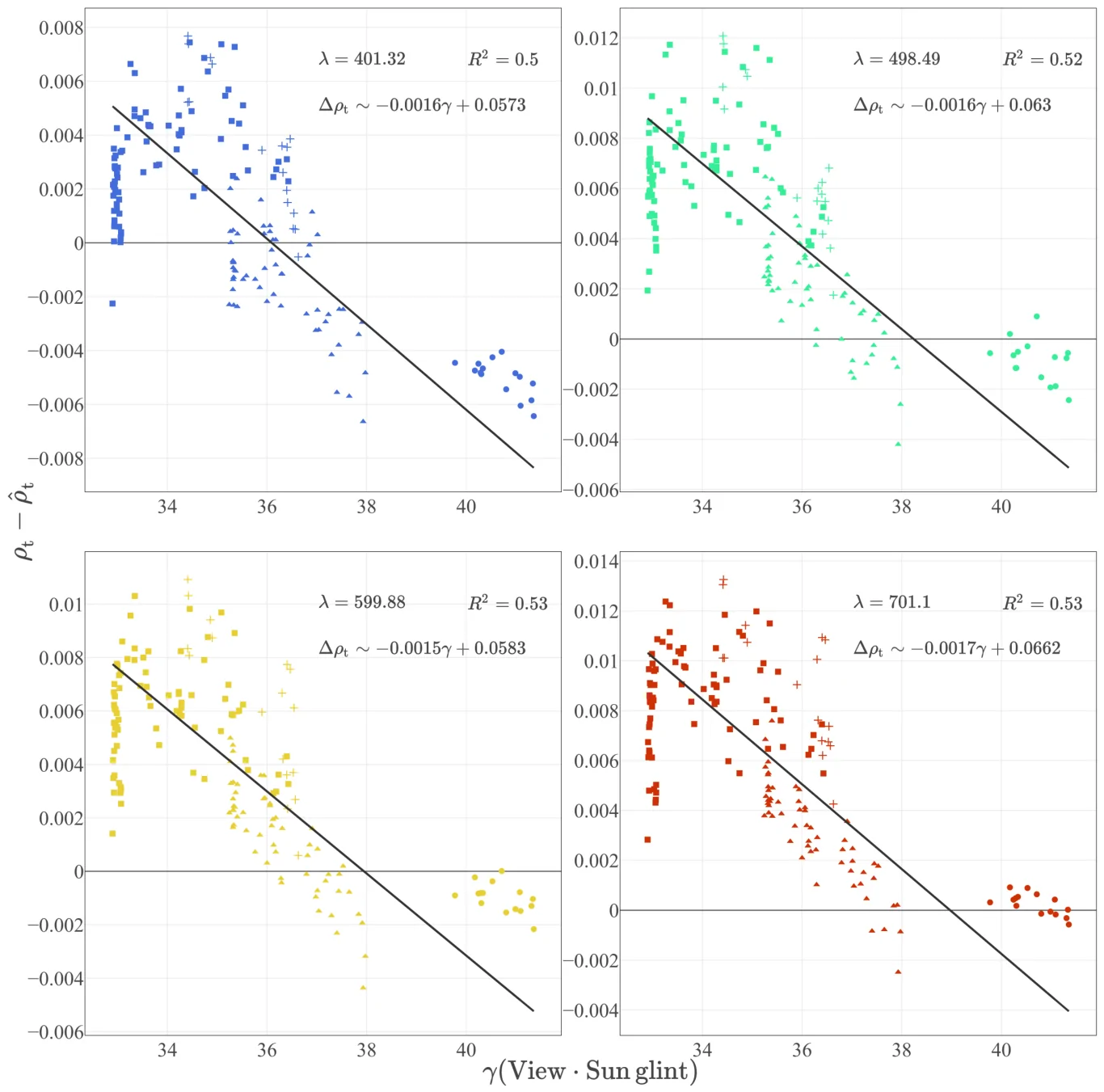
Relation between the residual of measured and modelled $\rho_{t}$ and angular distance of viewing direction from the sun’s specular reflection. Circles correspond to image ACI-11, triangles to image ACI-12, squares to image ACI-13, and cross to image ACI-14.

The mismatch between measured and modeled at-sensor reflectance derived from the jet-ski matchup dataset shows a strong dependence on viewing geometry relative to the specular direction (Figure 6). Specifically, the residual, $\Delta \rho_{t}=\rho_{t}-{\hat{\rho }}_{t}$, increases as the viewing direction approaches the specular reflection direction, consistent with the effect of sun-glint contributions that are not represented in the atmospheric correction model (these are only corrected after atmospheric correction, i.e., as in $\rho_{w}\left(G21\right)$).

**Figure 7:**
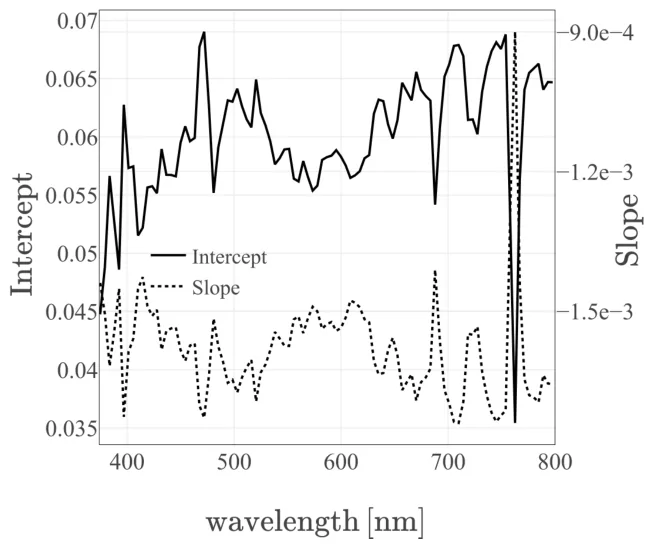
Spectral representation of the $\Delta \rho_{t}∼a\gamma +b$ model coefficients.

The linear dependence of $\Delta \rho_{t}$ on γ is spectrally flat (note the narrow range of slope values in Figure 7), indicating that the glint-driven residual behaves approximately as a wavelength-independent offset across the spectral range considered (similar to the atmospheric path radiance intercept in an EL calibration).

### 3.3. Ratio Calibration

**Figure 8:**
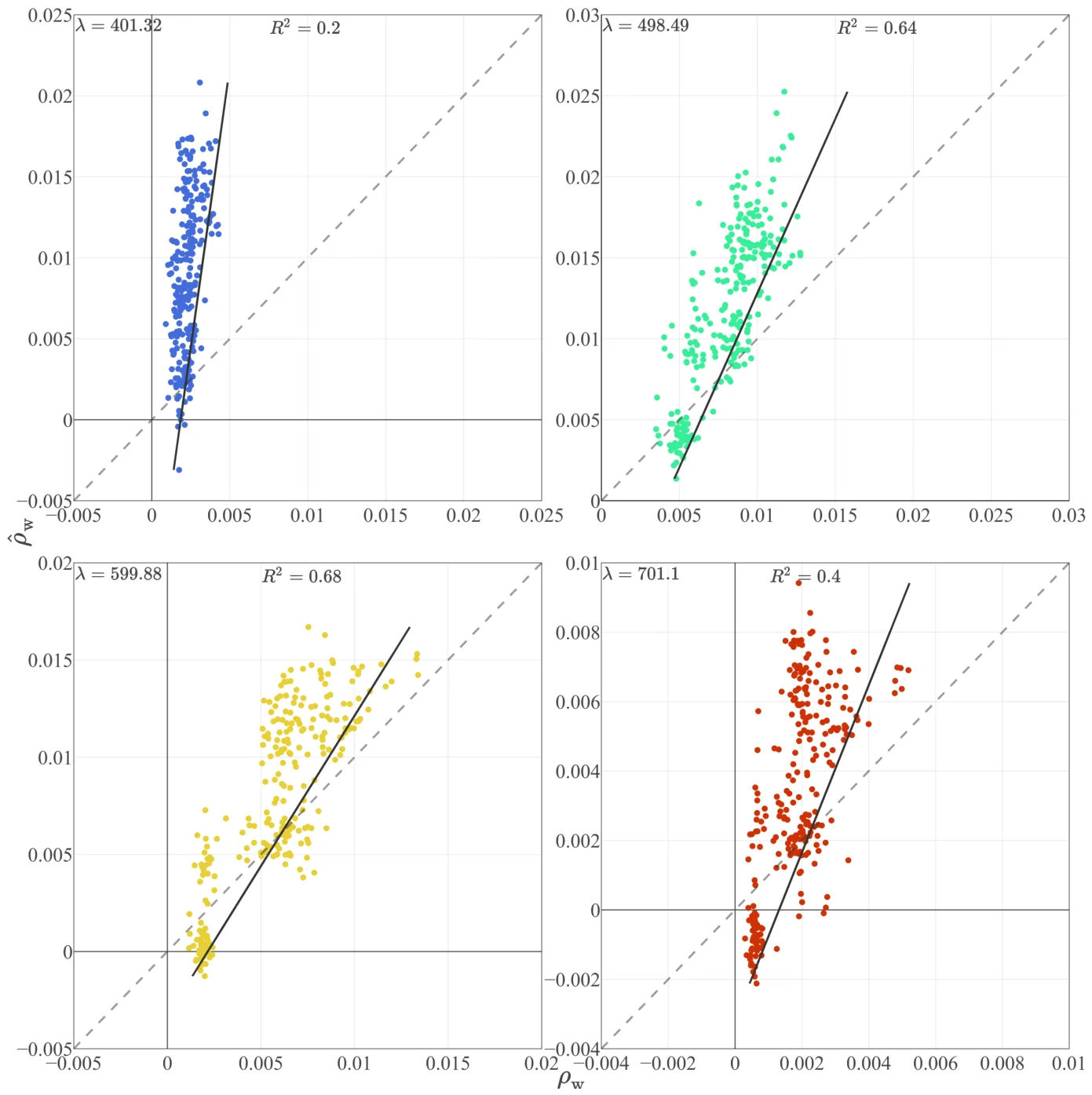
Water leaving reflectance retrieved from WISE Ratio SAS calibration (${\hat{\rho }}_{w}$) compared to in situ $\rho_{w}$ from the jet-ski at four wavelengths.

**Figure 9:**
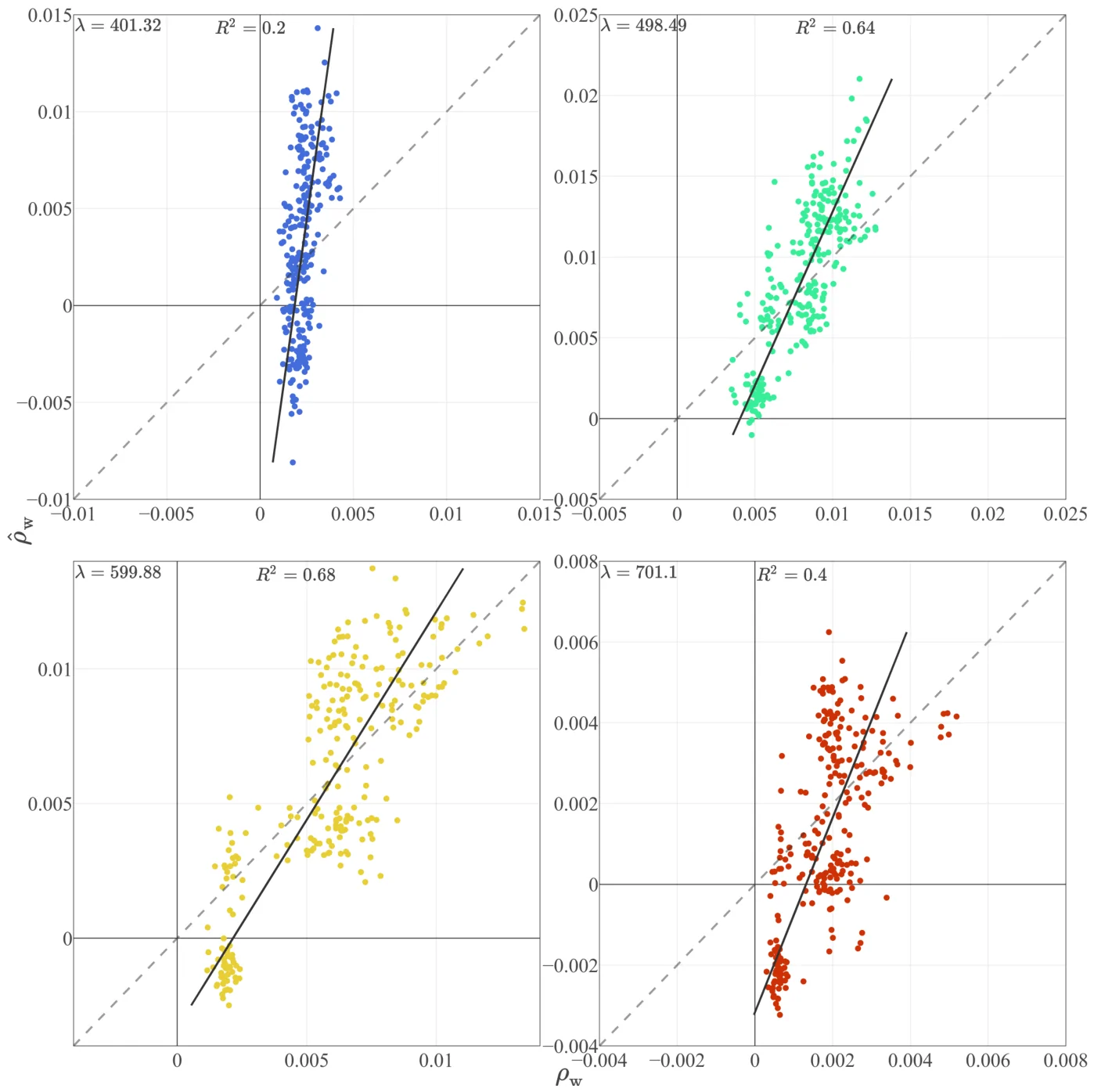
Water leaving reflectance retrieved from WISE Ratio SAS-jetski calibration (${\hat{\rho }}_{w}$) compared to in situ $\rho_{w}$ from the jet-ski at four wavelengths.

Applying the *Ratio* calibration derived from the SAS dataset gain to $\rho_{t}$ slightly improved the precision and accuracy at 400, 500, and 600 nm (Figure 8). However, for both the SAS-only and SAS-jetski calibration gains, the method fails to reproduce the lowest reflectances at 600 and 700 nm, where a substantial fraction of the retrieved ${\hat{\rho }}_{w}$ becomes negative. This issue is even more pronounced when using the *Ratio SAS-jetski* gains (Figure 9), making it the worst-performing approach. This highlights a key pitfall of vicarious-calibration strategies that do not account for the error structure of the input dataset or the assumptions inherent in the underlying model.

Applying the [18] sun-glint correction partially mitigates this problem, mainly by forcing negative reflectances near 800 nm to zero. Still, the *Ratio* approach remains the least robust method across the visible spectrum.

### 3.4. EL Calibration

**Figure 10:**
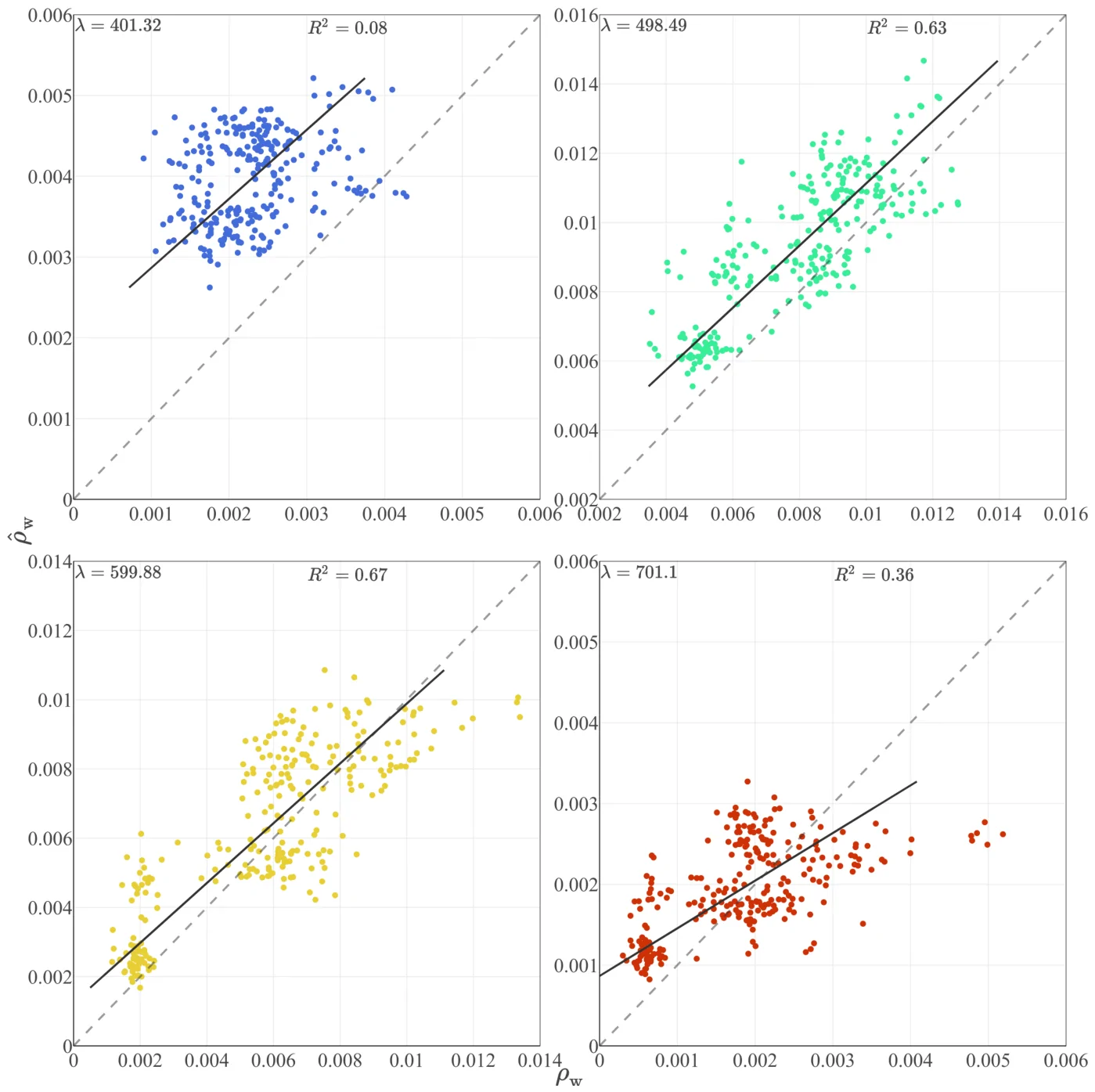
Water leaving reflectance retrieved from WISE EL OLS calibration (${\hat{\rho }}_{w}$) compared to in situ $\rho_{w}$ from the jet-ski at four wavelengths.

The EL calibration using OLS regression improves the blue-band retrieval relative to DSF pre-SVC, reducing the spread of ${\hat{\rho }}_{w}$ at 400 nm while maintaining a positive bias (Figure 10). At 500 and 600 nm, the dynamic range of both ${\hat{\rho }}_{w}$ and the observed $\rho_{w}$ is well reproduced, although a slight overestimation bias remains. At 400 nm, the retrieval is still consistently biased high, but the distribution is noticeably less dispersed relative to the *Ratio* method. In contrast, the retrieval at 700 nm shows a strong flattening of the WISE-derived ${\hat{\rho }}_{w}$, which does not follow the observed $\rho_{w}$ distribution. This results in lower accuracy at 700 nm compared with 500-600 nm, but performance remains comparable to that at 400 nm.

**Figure 11:**
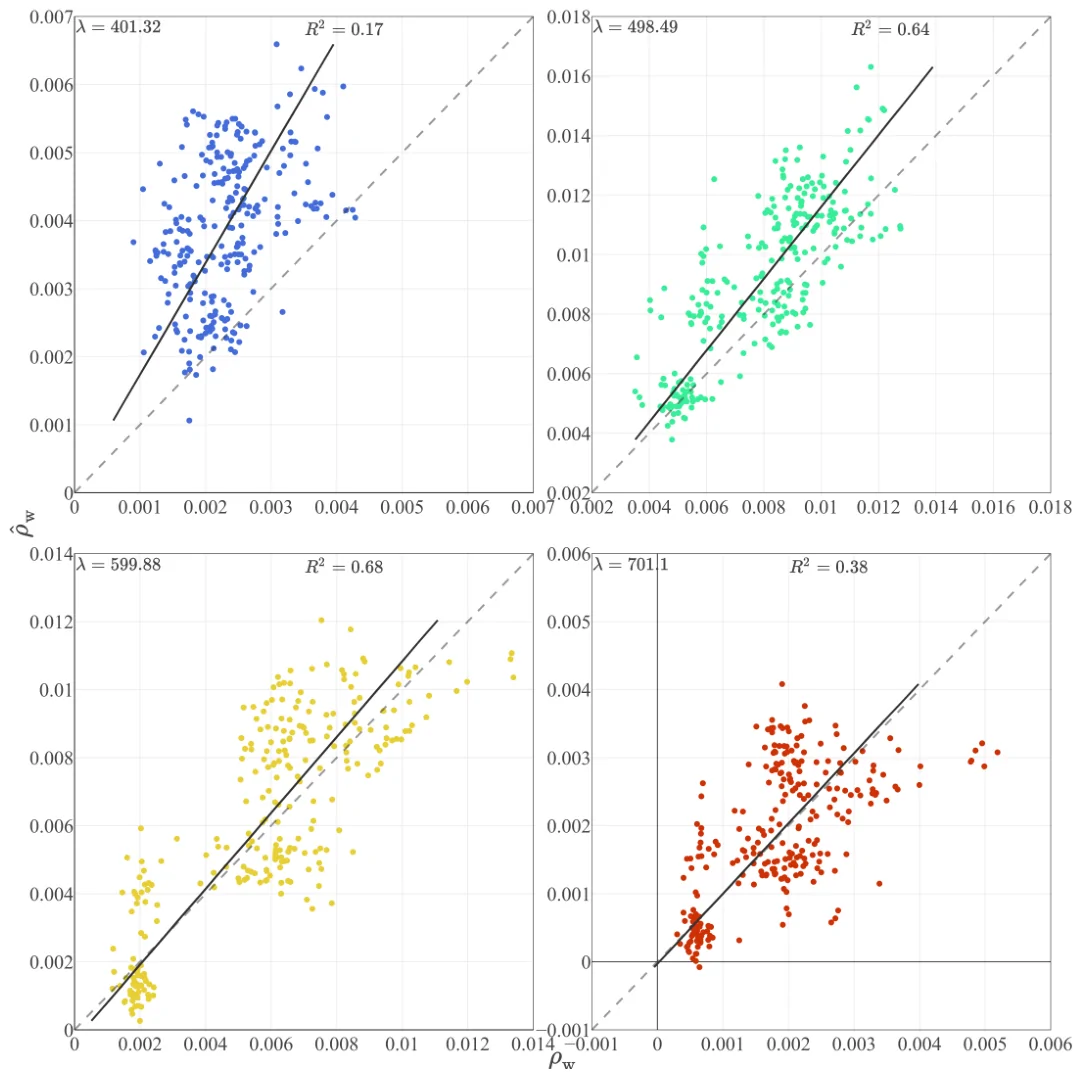
Water leaving reflectance retrieved from WISE EL SMA calibration (${\hat{\rho }}_{w}$) compared to in situ $\rho_{w}$ from the jet-ski at four wavelengths.

The EL calibration using Type II SMA regression provides the most consistent agreement with the in situ $\rho_{w}$ distribution across the visible range (Figure 11). In particular, the blue-band retrieval shows improved linearity while avoiding negative values. The mid-visible bands exhibit reduced systematic offsets compared to both the *Ratio* and *EL OLS* approaches. At longer wavelengths, *EL SMA* also mitigates the excessive flattening observed with *EL OLS*, yielding a more realistic spectral variability relative to the in situ $\rho_{w}$.

### 3.5. SVC Methods Comparison

**Figure 12:**
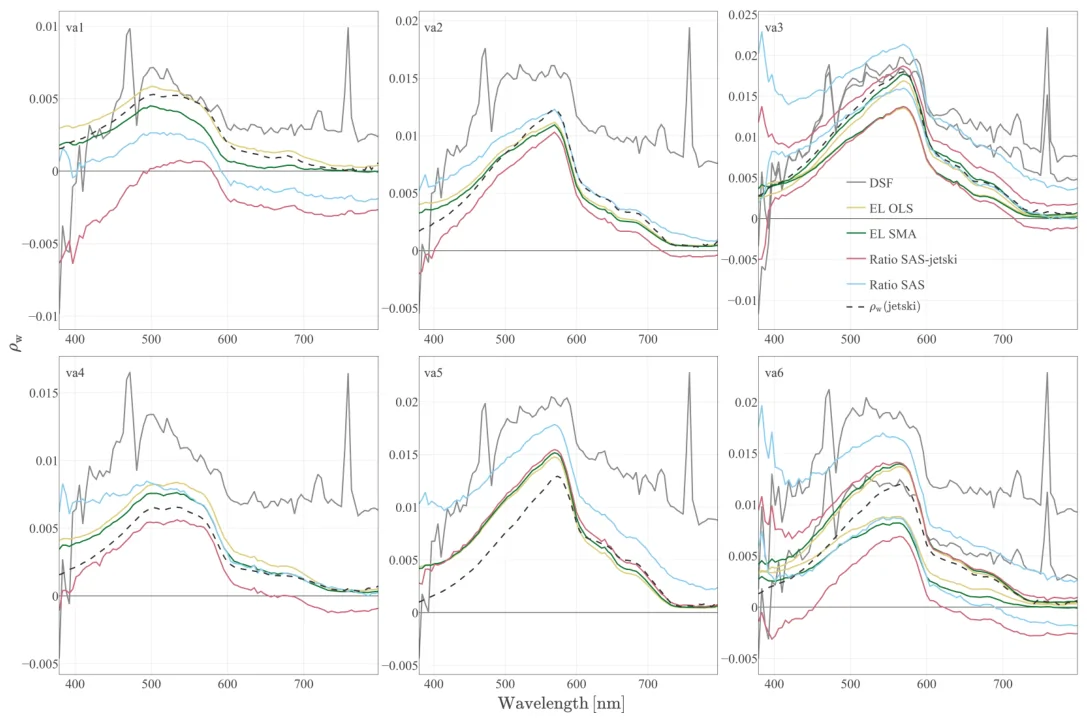
Water leaving reflectance for the six stations and five methods of ${\hat{\rho }}_{w}$ retrieval. Stations va3 and va6 have two flightline matchup for the same in situ ${\hat{\rho }}_{w}$.

We selected six in situ $\rho_{w}$ spectra (Figure 12) to form a representative set of validation stations across the initial DSF correction and four SVC approaches. The pre-SVC DSF retrieval shows the largest spectral distortion. The SVC methods reduce these distortions to varying degrees, with *EL SMA* providing the most plausible spectral shape and variability.

**Figure 13:**
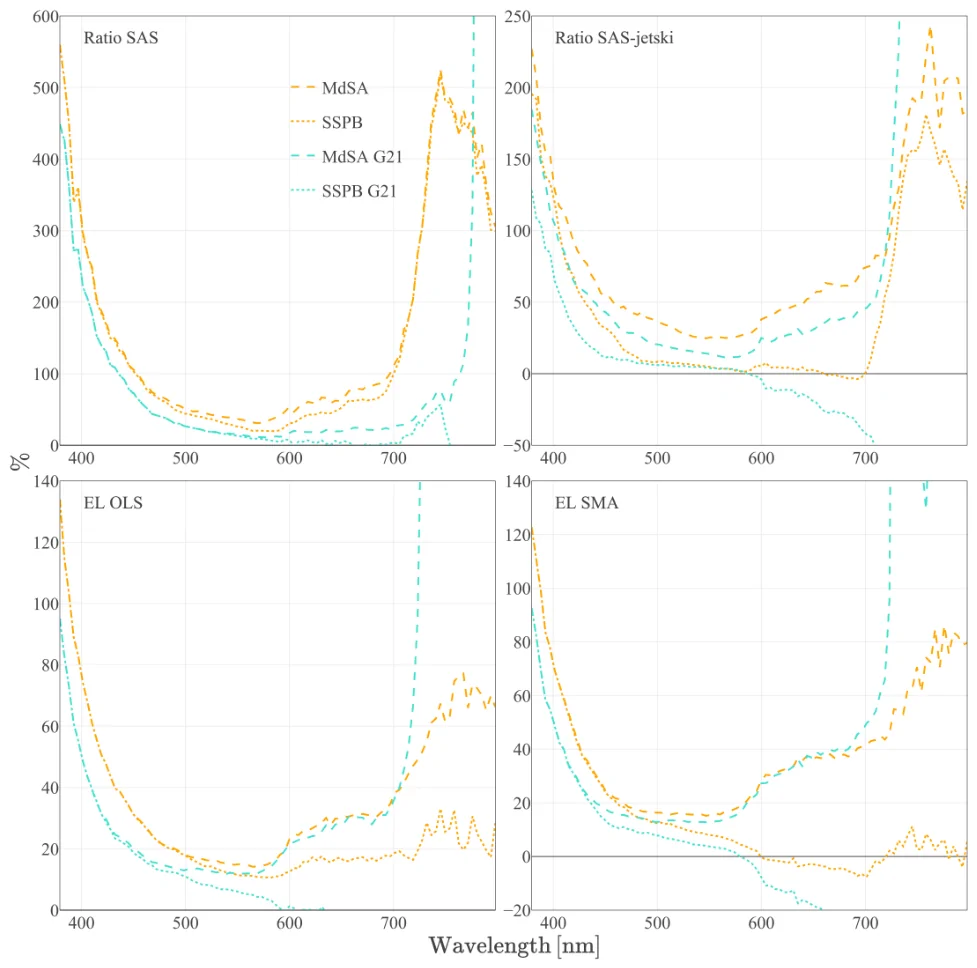
Median Symmetric Accuracy and Symmetric Signed Percentage Bias for the four vicarious calibration approaches.

**Figure 14:**
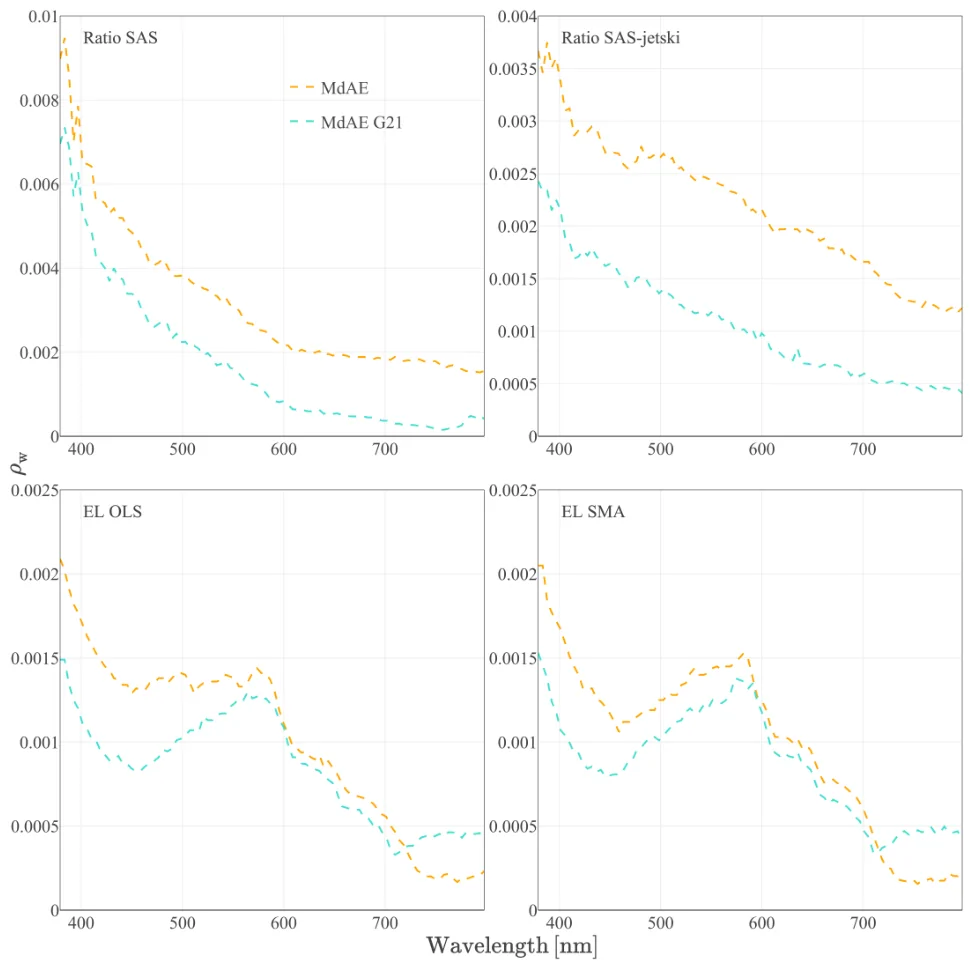
Median Absolute Error for the four vicarious calibration approaches.

We summarize performance using MdSA and SSPB (Figure 13), which together describe spectral precision and signed bias. In addition, we report the median absolute error in reflectance units (Figure 14), which is informative in low-$\rho_{w}$ conditions where relative metrics can become overly inflated. At 500 nm, the precision and bias of the *Ratio* method are ∼50%, while the *EL SMA* reaches ∼20%. Across all three metrics, $\rho_{w}$ *EL SMA* retrieval provides the best overall compromise, achieving both reduced bias and improved spectral consistency compared with other calibration approaches. Applying the sun glint correction following [18], applied after the *EL SMA* calibration, yields a modest improvement in precision and accuracy within the 400–700 nm range. However, this improvement comes at the cost of slightly degraded performance at wavelengths beyond 700 nm (Figure 13 and Figure 14).

## 4. Discussion

### 4.1. Pre-SVC Retrieval Noise

The DSF atmospheric correction applied to the pre-SVC WISE at-sensor reflectance produced water-leaving reflectance spectra that are generally overestimated and required the application of a sun glint correction [18]. This correction, in turn, led to negative values of ${\hat{\rho }}_{w}\left(G21\right)$ below 420 nm. This behavior is expected when small radiometric biases remain in $\rho_{t}$, because $\rho_{w}$ represents only a small residual of the total at-sensor signal after removal of atmospheric path and surface-reflected terms. In aquatic remote sensing, even a modest absolute bias in $L_{t}$ or in the modeled atmospheric term can therefore translate into a large error in $\rho_{w}$, particularly in the blue region, where Rayleigh scattering and aerosol contributions dominate [1,7]. Some of the noise seen in Figure 3A may arise from differences between the radiative-transfer model used for the initial in-flight radiometric calibration (MODTRAN with ATCOR) and the model used in this study (6S).

A second contributor is the sun-glint correction itself. The method of Gao and Li [18] enforces $\rho_{w}\left(800\right)\approx 0$ and subtracts a scaled Fresnel reflectance term. When the uncalibrated $\rho_{t}$ spectrum contains a wavelength-dependent bias (e.g., overestimated NIR and underestimated visible reflectance), the NIR normalization step propagates a systematic offset across the entire spectrum. These results reinforce the conclusion that, for airborne aquatic imaging spectroscopy, the atmospheric correction step must be coupled with a SVC procedure to achieve the radiometric accuracy required for quantitative applications [1].

### 4.2. Determination of Vicarious Calibration Gains

The gain spectra estimated from the *Ratio* and *EL* approaches demonstrate that derived calibration depends strongly on the structure of the matchup dataset and on unmodeled contributions in the forward model. In our case, the modeled–measured relationships at fixed wavelengths exhibit weak sensitivity (i.e., flat slopes) in portions of the spectrum (notably around 400 and 700 nm; Figure 4). This behavior is consistent with the combination of (1) a limited dynamic range in $\rho_{w}$ and (2) the dominance of atmospheric and surface-reflection terms in $\rho_{t}$ in these spectral domains.

When the matchup dataset is expanded (e.g., from HyperSAS-only to the merged HyperSAS-jetski dataset), the estimated gains change accordingly. This is expected, as the merged dataset spans a wider range of $\rho_{w}$ values and viewing/illumination geometries, thereby exposing model inadequacies such as unmodeled BRDF effects or sun-glint contributions. In practical terms, this means that gains derived from a narrow set of conditions can be internally consistent but fail to generalize beyond those conditions [23]. Conversely, incorporating a more diverse dataset can improve robustness—but only to the extent that the calibration model is capable of absorbing the additional variability. When this is not the case, broader datasets simply reveal structural mismatches between the model and the observed physics, leading to unstable gain estimates.

### 4.3. The Ratio Approach and Its Sensitivity to Sun-Glint

The *Ratio* method constrains the calibration to a purely multiplicative correction, equivalent to performing an OLS regression forced through the origin. This constraint is appealing when the mismatch is dominated by a single radiometric scaling factor. However, it becomes fragile when additive terms remain in the signal. Sun glint is such an additive term: it increases $\rho_{t}$ in a geometry-dependent manner and is not captured by our atmospheric correction model. When an additive contribution is present, a through-the-origin correction systematically distorts the estimated slope.

This behavior is consistent with the observed dependence of $\Delta \rho_{t}$ on the angular distance to the specular direction (Figure 6). Under these conditions, the *Ratio* approach tends to over-correct in the blue and red regions, where atmospheric-path terms dominate and water-leaving reflectance approaches zero. Because the *Ratio* method cannot absorb the additive sun-glint contribution, the resulting gain is structurally biased, leading to erroneous corrections across the spectrum. In short, the *Ratio* approach fails in the presence of unmodeled additive effects.

### 4.4. Empirical Line Method

The Empirical Line (EL) approach relaxes the constraint of the *Ratio* method by allowing both a multiplicative term (*a*) and an additive term (*b*) in the relationship ${\hat{\rho }}_{t}∼a\rho_{t}+b$. Conceptually, the additive term can absorb residual offsets associated with path radiance and unmodeled surface-reflected contributions (e.g., sun glint), while the multiplicative term adjusts radiometric scaling. This makes EL better suited to situations where the forward model is imperfect or where the data contains effects not explicitly accounted for. In classical EL formulations, the intercept is often interpreted as an estimate of atmospheric path radiance. In our study, the intercept term represents a mixture of misrepresented atmospheric path radiance [5] and residual sun-glint contributions. Previous studies have noted that the EL approach (and vicarious calibration more generally) can introduce unexpected uncertainties [23] if the characteristics of the calibration dataset are not carefully considered.

Within the EL framework, the choice of regression estimator is critical. Ordinary Least Squares (OLS) assumes that the predictor is measured without error, which was not the case in our experimental setup in a natural environment: both the measured $\rho_{t}$ and the modeled ${\hat{\rho }}_{t}$ carry uncertainty arising from $\rho_{w}$ inputs, atmospheric-correction steps, and unmodeled effects such as residual sun glint. Standard Major Axis (SMA) regression (Type II) provides a symmetric treatment of variability and uncertainty in both axes and is therefore more appropriate when both variables include measurement and modeling errors [21]. Our results demonstrate the use of the *EL SMA* method for the retrieval of $\rho_{w}$ with an accuracy improving from 80 to 20% between 400 and 460 nm, stable at 20% between 460 and 580 nm, decreasing from 20 to 40% between 580 and 700 nm, and decreasing from 40 to 80% from 700 to 800 nm (Figure 13). In our results, SMA produces a calibration that is more spectrally stable (i.e., behaves similarly across the spectrum instead of improving only the retrieval of some wavelengths) and improves agreement in the blue region, without generating the degree of spectral flattening observed with OLS at longer wavelengths (Figure 10 and Figure 11).

### 4.5. Implications for Aquatic Remote Sensing Application

For aquatic applications, preserving the spectral shape of $\rho_{w}$ is often as (if not more) important as reducing magnitude errors. Many bio-optical and shallow-water semi-analytical inversion methods (e.g., [24,25,26,27]) rely on spectral slopes, curvature, and band ratios. As a result, a calibration that reduces bias at one single wavelength but distorts the overall spectral shape can degrade the retrieval of water constituents and bottom-related signals. The pre-SVC DSF retrieval illustrates this risk: negative blue reflectances, spectral noise, and wavelength-dependent offsets (Figure 2 and Figure 3) would directly propagate into errors in the interpretation of absorption-driven features and into any inversion algorithm.

The spectrum of the EL intercept is also informative: it follows a blue-to-red decrease consistent with the expected behavior of atmospheric-path and surface-reflected contributions, supporting the interpretation that additive terms remain in $\rho_{t}$. Allowing a nonzero intercept is therefore necessary when an unaccounted additive component—such as sun glint—is present. In this context, the *EL SMA* calibration provides the best compromise between reducing bias and maintaining a physically plausible spectrum, yielding $\rho_{w}$ suitable for quantitative aquatic remote sensing and for use as input to optically shallow-water inversion workflows. A key issue is the relationship between the residual structure of $\Delta \rho_{t}$ and the angular distance to the sun’s specular reflection. This dependence may be partially confounded with the bidirectional reflectance distribution function (BRDF) of the water-leaving reflectance, particularly in optically shallow waters [28]. In such conditions, angular effects in bottom-reflected light can interact with surface glint geometry, complicating attempts to attribute residuals to a single cause.

The observed relationship between $\Delta \rho_{t}$ and the angular distance from the sun’s specular point, γ, (Figure 6) indicates that sunglint remains a primary source of mismatch between modeled and observed at-sensor reflectance in the matchup dataset. This outcome was expected, given that moderate to strong southwesterly winds were blowing steadily during the field campaign, affecting the quality of both the airborne WISE measurements and the in situ radiometry. The glint-related residual behaves predominantly as an additive term, and its fitted angular dependence is spectrally flat (Figure 7), consistent with sunglint acting as a broadband contribution across the visible and NIR spectral range.

This result can be interpreted in the context of the sunglint-correction method of [18], which incorporates the wavelength dependence of Fresnel reflectance. Although this spectral dependence is physically correct, it is small compared to other sources of variability, including wave-slope statistics, adjacency effects, imperfect aerosol characterization, and measurement noise. As a result, the spectral imprint of sunglint is difficult to isolate empirically in matchup analyses.

A practical implication is that vicarious calibration strategies should explicitly consider glint geometry, either by restricting matchups selection to low-glint conditions or by introducing an additional term capable of absorbing glint-related variability. Without such measures, the calibration may inherit geometry-dependent biases that propagate into downstream aquatic products.

## 5. Concluding Remarks and Recommendations

Residual sun glint behaves as a spectrally flat additive component in $\rho_{t}$ and systematically biases multiplicative vicarious calibration. For this reason, the sun-glint contribution should be incorporated directly in the atmospheric correction model itself (rather than corrected afterward) following approaches similar to [29] or [10]. Because a purely multiplicative correction (Ratio) is inherently unstable in the presence of additive terms, an Empirical Line method that includes an intercept is recommended whenever residual additive contributions are expected (e.g., sunglint, imperfect modeling of atmospheric path radiance).

When both modeled and measured quantities contain uncertainty, the Standard Major Axis gives better results than the Ordinary Least Squares for the EL fit. In the absence of a reliable glint correction incorporated in the atmospheric correction model, matchups should be filtered or weighted based on a glint distance metric (e.g., γ) to avoid geometries close to the specular direction. Even without those precautions, the EL SMA system’s vicarious method provides data of sufficient quality for application in optically shallow water remote sensing. This makes *EL SMA* a robust and practical choice for airborne aquatic imaging spectroscopy under realistic field conditions.

Looking forward, the results of this study underscore that rigorous radiometric and vicarious calibration is not an optional refinement but a foundational requirement for the reliable use of airborne and drone-based hyperspectral imagery in aquatic environments. As compact hyperspectral sensors become increasingly available—including systems suitable for deployment on drones, AUVs, and other lightweight platforms—the temptation to treat them as “plug-and-play” instruments is growing. Yet the physics does not change: accurate retrieval of water-leaving reflectance still depends critically on careful correction of atmospheric effects, sensor-specific radiometric biases, and geometry-dependent contributions such as sun glint. Without such calibration work, downstream products—whether related to water-column biogeochemistry, shallow-water mapping, or ecological monitoring—are at risk of inheriting unquantified and potentially misleading errors. Ensuring that future HS systems integrate robust calibration strategies, either through improved forward modeling or through well-designed vicarious calibration workflows, will be essential for the next generation of aquatic remote-sensing applications.

## Data Availability

The data that support the findings of this study are openly available from the Saint-Lawrence Global Observatory (SLGO) repository at https://doi.org/10.26071/72f6c659-7383-4ff4. The software developed for this work is published under the GPLv3 license on GitHub at https://github.com/raphidoc/aabim (accessed on 4 September 2026).

## List of Abbreviations and Symbols

ATCOR	Atmospheric and Topographic Correction
AVIRIS	Airborne Visible/Infrared Imaging
CASI	Compact Airborne Spectrographic Imager
DSF	Dark Spectrum Fitting
EL	Empirical Line
HOCR	Hyperspectral Ocean Color Radiometer
HyperSAS	Hyperspectral Surface Acquisition System
ITRES	Infrared and Thermal Research Limited
LUTs	Look-Up Tables
MdSA	Median Symmetric Accuracy
MdAE	Median Absolute Error
OLS	Ordinary Least Squares
PRISM	Portable Remote Imaging Spectrometer
SMA	Standard Major Axis
SSPB	Symmetric Signed Percentage Bias
SVC	System Vicarious Calibration
WISE	Watersat Imaging Spectrometer Experiment
$\rho_{t}$	At-sensor reflectance
$\rho_{w}$	Water-leaving reflectance
γ	angle between the viewing direction and the sun’s specular reflection direction
